# Peritonitis from Perforation, with Uncommon Symptoms

**Published:** 1855-08

**Authors:** Lewis Rogers

**Affiliations:** Professor of Materia Medica and Therapeutics in the University of Louisville


### The Western Journal

OF

### MEDICINE AND SURGERY.

AUGUST, 1855. NEW SERIES. VOL. IV.—NO. 2.

### ART. I.—PERITONITIS FROM PERFORATION, WITH UNCOMMON SYMPTOMS.

BY LEWIS ROGERS, M. D., PROFESSOR OF MATERIA MEDICA AND THERAPEUTICS IN THE UNIVERSITY OF LOUISVILLE.

ACUTE PERITONITIS is one of the modes of fatal termination of typhoid fever. It may be caused by the progressive extension of the peculiar intestinal lesion so constantly present in this affection, to the peritoneal coat, without a solution of continuity of the bowels, but, in most instances, it results from ulcerative perforation of the intestine and the effusion of its contents into the cavity of the peritoneum. The symptoms which announce this very formidable event are usually strongly marked, and sufficiently distinctive in character and mode of onset to be clearly diagnostic of the precise nature of the accident. Besides such as are indicative of a great shock, sustained by the nervous and circulatory systems, and by the vital powers generally, observers describe two classes of symptoms of a local kind, pertaining to the abdomen and urinary organs, those connected with the viscera strictly abdominal being almost invariably present, while those associated with the urinary apparatus are only occasionally observed. In addition to these two classes of symptoms, it has occurred to me to notice several other symptoms attendant upon perforation, which must have escaped previous observation, as I cannot find them alluded to in any of the works upon the subject.

The abdominal symptoms of perforation are acute pain *suddenly* developed in some region of the abdomen, extending with great rapidity to every part of it; exquisite tenderness on pressure, beginning at the point of perforation and diffusing itself generally over the entire cavity; hard tympanitic distension; nausea and vomiting. The pain of perforation is exceedingly severe, persistent, commonly insusceptible of any thing more than partial mitigation, and continuing unsubdued until sensibility is lost in exhaustion.

I have remarked, and with a purpose, which will presently appear, that the symptoms are usually so clear and distinctive that there is rarely any obscurity in the diagnosis. LOUIS, whose observatons on this subject are most accurate and extensive, states, that—"if in an acute disease, and in an unexpected manner, a violent pain of the abdomen suddenly supervenes, if this pain is exasperated by pressure, accompanied by rapid alteration of the features, and more or less promptly followed by nausea and vomiting, we may believe and announce that there is a perforation of the intestine."

The symptoms of perforation, ordinarily so frank, are not, however, invariably so. This fact deserves attention as a mere matter of correct diagnosis, if not of practical importance. I have reason to think that perforation occurred, without well-marked abdominal symptoms, in one of the cases which will be briefly reported presently.

To cases of masked or latent perforation, Dr. STOKES adverts, in his article upon Peritonitis in the Cyclopœdia of Practical Medicine. He says: "It is a fact, however, that, in a very few cases, this accident may supervene without the ordinary sudden and violent symptoms: thus, in the ninth observation of LOUIS, we read of a patient, who was laboring under fever with diarrhœa, in whom the symptoms which appeared to correspond with the perforation were delirium and shivering, which occurred in the morning and continued till the evening of the next day, when the patient sank; there was also increased meteorism, and the patient winced only after a strong pressure of the belly. In this case the perforation was not suspected, yet LOUIS inquires whether we might not, under similar circumstances, suspect a perforation of the intestines, particularly when it is considered that peritonitis without perforation is rare as occurring in the course of acute diseases. He thinks that if, in the course of a continued fever with diarrhœa, the patient is seized with delirium, shiverings, and a slight tenderness of the belly, which until then had not been painful, we might be authorized to suspect a perforation of the small intestine."

Disturbance of the urinary organs is not an uncommon occurrence in the peritonitis produced by perforation of the bowels; it is also a frequent symptom of simple peritonitis. This disturbance consists in a constant, and painful desire to urinate, and in an inability, suddenly developed, to evacuate the bladder; this organ is frequently found empty and its mucous membrane slightly inflamed. The urinary symptoms are generally associated with and secondary to those of the abdomen, though Dr. Stokes reports three cases, in which irritation of the bladder was the *first* symptom observed. In simple peritonitis, painful and unsuccessful efforts at micturition frequently greatly annoy the patient; the retention often demands the use of the catheter. The inflamed peritoneal investment of the bladder seems to exert a paralyzing influence upon the muscular coat of this organ, and at the same time, to exalt the sensibility of the mucous coat. An effect is produced upon the bladder analogous to that which exists in the intestinal canal, the peristole of which is always retarded, and constipation induced, often so obstinate, as to resist the most active cathartics.

The symptoms of perforation, thus far considered, have been noticed by most observers, and are described more or less fully by writers upon peritonitis. A third class of symptoms have presented themselves to me, connected with the organs of generation. In the first case in which one of these symptoms was manifested, I looked upon it as an accidental phenomenon, having no special or positive connection with the other symptoms of the case; but something analogous having been observed in several other patients, not only by myself, but by others, who were not apprised of my observation, I have been forced to the inference, that the association is not merely casual. The new symptoms, which I have observed, are intense venereal passion suddenly developed, priapism, and intolerable pain in the penis. Three of the cases in which one or more of these symptoms existed are defective, for the reason, that the existence of perforation was not ascertained by post-mortem examination; the other two are complete in this particular.

W. N. M., aged twenty-seven, was the subject of typhoid fever in the spring of 1848; the attack was moderately severe, and protracted; convalescence seemed, however, to be ultimately established, and an opinion to this effect was given by the attending physician, on the afternoon of Friday, May 5th. Shortly after the evening visit of the physician, Dr. M. was suddenly seized with intense and irresistible venereal desire; the gratification of this desire was immediately succeeded by violent pain in the abdomen; all the characteristic symptoms of peritonitis from perforation were at once developed, and death ensued on the afternoon of May 6th, about twenty-four hours after the seizure. A post-mortem examination was not made, and in this respect the case is incomplete, and unsatisfactory, but the symptoms were so strongly marked, and developed in such a manner, as to leave scarcely a doubt as to the nature of the lesion. Receiving as correct, the diagnostic marks of perforation as propounded so positively by Louis in the quotation made above, it seems to me that an autopsy was scarcely requisite, to assure the correctness of the diagnosis in this case.

R. D. M., aged twenty-seven was the subject of typhoid fever in Jan. 1854; the attack commenced insidiously early in the month, and, in its progress, presented most of the symptoms, which characterize this form of disease; subsultus tendinum, occasional delirium and sopor existed; diarrhœa, with a dry, brown tongue, and slight abdominal tenderness were present; pulse frequent and feeble, and muscular prostration considerable. On Friday night, January 27th, strong venereal desire was suddenly developed and was gratified; on Saturday, the nervous symptoms were much aggravated, the delirium being now constant and complete; muscular prostration greater and abdominal tenderness increased; pulse very frequent, and countenance hippocratic; no shiverings were noticed. These symptoms grew rapidly worse, and death ensued on Sunday night, about forty-eight hours after the sudden development of venereal desire. A post mortem examination was not obtained in this case, yet I feel quite well satisfied, that it was one of masked or latent perforation, such as Dr. Stokes describes in the quotation made from his paper. The decided abdominal symptoms, usually present, were not disclosed, because of the existence of excessive disturbance of the nervous system; this disturbance was very materially aggravated during the last two days, the abdominal tenderness somewhat so, but not sufficiently to justify a suspicion of perforation.

This marked condition of disease is observed, we know, in other grave affections. The heart and its membranes are occasionally the subjects of intense and fatal inflammation, without the fact being disclosed by any rational sign pertaining to this organ; to the mind of the intelligent physician, however, the peculiar disturbance of the nervous system, which wholly masks that of the heart, is a pregnant fact, and auscultation reveals to him the real and primary seat of disease.

The occurrence of strong venereal passion, in the last stage of typhoid fever, is certainly a very remarkable phenomenon; whether a sequence of perforation or not, as a matter of fact it is an extraordinary symptom, and must have some corresponding mode of causation.

Major P. B., aged fifty-five, in January, 1851, after an illness of an uncertain and ill-defined character, of several weeks duration, was suddenly seized, on the night of the 14th, with violent abdominal pain. I saw him in consultation with Dr. E. early in the morning of the 15th; he was then suffering with intense pain over the entire abdomen, accompanied by exquisite tenderness on pressure, and with pain, still more intense in the penis; pulse frequent and compressed, countenance somewhat pinched and hippocratic, and vital powers greatly depressed. Though the pain of the abdomen had all the characteristic severity of that which attends acute peritonitis, it was incomparably less than that in the penis; this extracted from the patient, who was a gentleman of more than ordinary firmness, expressions of the most intolerable anguish; it was, in fact, terrific. These symptoms were but partially mitigated by the very liberal use of opium, and were present at 9 o'clock, when I made my last visit to him; he died in the course of the night, and a post-mortem examination, on the following day, disclosed the usual appearances of peritonitis from perforation, a single perforating ulcer existing in the lower part of the ileum, through which, castor oil and other matters, contained in the bowel, had escaped into the peritoneal sac.

This patient had been vaguely indisposed for several weeks before the supervention of his severe symptoms; he had never been sick enough to be confined constantly to bed, nor did he have, in a marked form, any of the symptoms which are usually present in typhoid fever; this, however, was the diagnosis of his attending physician, and he was no doubt correct, the case corresponding in its severity with those cases of typhoid fever, in which perforation has been observed most frequently to occur. In reference to this point Dr. BARTLETT remarks—"In a majority of instances, it takes place in cases of moderate severity, and in those which have been described as *latent*, and at a late period of the disease."

George W. W., aged twenty-two, took to his bed November 17, 1854, and was brought up from Larue county, a distance of sixty miles, and placed under my medical charge November 19th. How long he had been complaining before going to bed I could not learn; he was one of a large family, nine members of which had typhoid fever, five of the nine cases proving fatal, several of them having hemorrhage from the bowels. The first of the series was that of Jacob W., who was attacked July 4th, and confined to his bed and room for two months; he was under my medical care part of the time, and had profuse and nearly fatal hemorrhage from the bowels. The father and mother came up from Larue county to visit this young man, and on their return home had the fever; the father sickened August 10th, and died October 17th; the mother October 17th, and died November 2d; she had hemorrhage for two days before her death. Elizabeth J. W., aged seventeen, was attacked September 20th, and died October 16th; had hemorrhage from the bowels. Mrs. Sarah H. T., a sister, aged twenty-seven, was attacked October 10th, and died November 15; suffered much with her bowels but did not have hemorrhage. Charles D. W., aged nineteen, was attacked October 4th, and recovered after a confinement to the house of six weeks; had considerable hemorrhage from the bowels. John H.

W., aged twelve, was attacked September 26th, and confined six weeks; recovered without hemorrage. Matilda W., aged fourteen, was attacked October 1st, and confined seven weeks; had slight hemorrhage from the bowels. George, the immediate subject of this notice, came under my charge November 19th and presented the usual symptoms of continued fever, until the morning of the 28th; at 10 o'clock that morning I was sent for and found him suffering most acutely with pain in the abdomen, which had suddenly seized him; excessive tenderness on pressure, and tympanitic fullness accompanied the pain; none of these symptoms existed at my visit early in the morning, and they were reported to have come on with great rapidity; counteuance much altered and expressive of suffering; pulse small and frequent; skin moist and cold. Warm fomentations were ordered to the abdomen, and full doses of morphia to be administered internally every hour until the pain was materially mitigated. I saw him several times during the day; the pain was only partially relieved by the morphia; at 6 o'clock in the evening he called my attention to a pain in the penis, which he said had existed for several hours; it was very severe, not so intense and dominant as that of which Major B. complained, yet sufficient to excite serious complaint even in the midst of the great abdominal suffering. This pain continued at 10 o'clock, P. M., the hour of my last visit; he died at 6 o'clock the following morning. The body was immediately taken to his native county for burial, and an autopsy thus prevented, but I have not the slightest doubt as to the character of the lesion, perforation of the bowel, which gave rise to the formidable array of symptoms preceding death.

Dr. William R. Evans, of Mercer county, Ky., an intelligent graduate of the University of Louisville, has kindly furnished me with the notes of the following case:

Thomas Norton, aged about thirty-five years, of Mercer county, Ky., a farmer by occupation, was attacked in April, 1852, with typhoid fever. For five or six days after the accession of the disease he continued to go about, though suffering very much from headache. There was no material change in his condition, from the last of the first week, about which time he took his bed, to the commencement of the third week, when he had pretty copious hemorrhage from the bowels; more or less blood was discharged for the two succeeding days, when he suddenly complained of a burning sensation in his bowels, with most intense pain in the course of the penis, especially at the head of this organ; this symptom was attended with priapism. He died in about twelve hours from the occurrence of these symptoms; a post mortem examination revealed perforation of the ileum, with extensive peritonitis. There was a constant desire to urinate, but no urine was voided, and on examination none was found in the bladder.

